# Complementation of a phycocyanin-bilin lyase from *Synechocystis *sp. PCC 6803 with a nucleomorph-encoded open reading frame from the cryptophyte *Guillardia theta*

**DOI:** 10.1186/1471-2229-8-56

**Published:** 2008-05-16

**Authors:** Kathrin Bolte, Oliver Kawach, Julia Prechtl, Nicole Gruenheit, Julius Nyalwidhe, Uwe-G Maier

**Affiliations:** 1 Philipps-Universität Marburg, Laboratorium für Zellbiologie, Karl-von-Frisch Str., D-35032 Marburg, Germany; 2 Heinrich-Heine Universität Düsseldorf, Institut für Botanik III, Universitätsstr. 1, D-40225 Düsseldorf, Germany; 3 Philipps-Universität Marburg, Laboratorium für Parasitologie, Karl-von-Frisch Str., D-35032 Marburg, Germany

## Abstract

**Background:**

Cryptophytes are highly compartmentalized organisms, expressing a secondary minimized eukaryotic genome in the nucleomorph and its surrounding remnant cytoplasm, in addition to the cell nucleus, the mitochondrion and the plastid. Because the members of the nucleomorph-encoded proteome may contribute to essential cellular pathways, elucidating nucleomorph-encoded functions is of utmost interest. Unfortunately, cryptophytes are inaccessible for genetic transformations thus far. Therefore the functions of nucleomorph-encoded proteins must be elucidated indirectly by application of methods in genetically accessible organisms.

**Results:**

Orf222, one of the uncharacterized nucleomorph-specific open reading frames of the cryptophyte *Guillardia theta*, shows homology to *slr*1649 of *Synechocystis *sp. PCC 6803. Recently a further homolog from *Synechococcus *sp. PCC 7002 was characterized to encode a phycocyanin-β155-bilin lyase. Here we show by insertion mutagenesis that the *Synechocystis *sp. PCC 6803 *slr*1649-encoded protein also acts as a bilin lyase, and additionally contributes to linker attachment and/or stability of phycobilisomes. Finally, our results indicate that the phycocyanin-β155-bilin lyase of *Synechocystis *sp. PCC 6803 can be complemented *in vivo *by the nucleomorph-encoded open reading frame *orf*222.

**Conclusion:**

Our data show that the loss of phycocyanin-lyase function causes pleiotropic effects in *Synechocystis *sp. PCC 6803 and indicate that after separating from a common ancestor protein, the phycoerythrin lyase from *Guillardia theta *has retained its capacity to couple a bilin group to other phycobiliproteins. This is a further, unexpected example of the universality of phycobiliprotein lyases.

## Background

Phycobiliproteins are subunits of the major accessory light-harvesting complexes (LHC) of most cyanobacteria and red alga and are present in the thylakoid lumen of cryptophytes as well. Covalently linked to the proteins are chromophore groups, the phycobilins [1,2]. These open tetrapyrrole rings are coupled to conserved cysteine residues via a thioether bond and are necessary for light harvesting and efficient energy flow [3]. Various phycobiliproteins, namely allophycocyanin, phycocyanin, phycoerythrin, phycoerythrocyanin, carry different numbers of bilin groups.

Attachment of bilins to phycobiliproteins is an enzymatically catalyzed reaction, which also occurs spontaneously, but at low efficiency [4]. Several bilin-attaching lyases are described. One of the dimeric enzymes encoded by *cpc*E and *cpc*F genes links the chromophore to the phycocyanin α-subunit [4,5]. *Pec*E and *pec*F genes encode the second known lyase, specific for the phycoerythrocyanin α-subunit [6-8]. Recently Zhao and co-workers discovered that a CpeS-like protein functions as a phycocyanobilin-cysteine-beta84 lyase in *Anabaena *sp. PCC 7120, which was the first lyase identified for a β-subunit of a phycobiliprotein [9]. Another lyase specific for a β-subunit of a phycobiliprotein was found by Shen et al. [10]. They identified the gene product of *cpcT *to be a Cys-β153-phycocyanobilin lyase in *Synechococcus *sp. PCC 7002. Moreover, Zhao et al. reported the *Anabaena *sp. PCC 7120 CpeS1 as a "near-universal" lyase for cysteine-84-binding sites in cyanobacterial phycobiliproteins [11,12].

In most cyanobacteria and red algae phycobiliproteins are organized in multimeric complexes, called phycobilisomes [13-15]. Their antenna structure, located on the cytoplasmic surface of the thylakoid membrane, consists of various linker polypeptides and phycobiliproteins. Each phycobilisome is on its part a multimeric complex, composed of a core and several rod structures. Phycobilisomes can be subdivided according to their structure. The most common type in cyanobacteria, the hemidiscoidal one, consists of a tricylindrical core and six rods.

Allophycocyanin (AP, λ_max _= 650 nm) forms the core structure, connecting the phycobilisomes to the thylakoid membrane via linker proteins. Rods can be composed of three different phycobiliproteins: phycocyanin (PC, λ_max _= 617 nm) is located proximal to the core, whereas phycoerythrin (PE, λ_max _= 560 nm) and phycoerythrocyanin (PEC, λ_max _= 575 nm) are located distal to the core [16,17]. The phycobilisome rods of each organism differ in their phycobiliprotein composition. *Synechocystis *sp. PCC 6803 harbors hemidiscoidal phycobilisomes. PC, the only biliprotein in the rod structures in this organism, is composed of α- and β-subunits. These subunits dimerize to heterodimers, assemble to hexameric (αβ)_6 _discs, and are subsequently coupled to each other, as well as to the AP-core via linker proteins [18,19]. Depending on their location (in core or rods), and their molecular mass, linker proteins are divided into four groups [20,21]. Beside their main function of mediating the assembly and stability of the phycobilisomes, linker proteins also promote energy transfer towards the reaction centres [20].

*Guillardia theta *is a cryptophyte possessing phycoerythrin as a phycobiliprotein. The β-subunit is encoded on the plastid genome [22], whereas the phycoerythrin α-subunits are encoded by a nuclear-located gene family. In the latter case, the genes encode preproteins containing a tripartite topogenic signal responsible for the translocation across five biological membranes [23]. Because a wide range of genomic data exists from this unicellular phototrophic organism, existing knowledge can be used to reconstruct the biochemistry of these organisms. The elucidation of protein functions encoded by open reading frames in the nucleomorph genome of *Guillardia theta *is of special interest, as this genome is minimized and should therefore encode only essential proteins. After analyzing the nucleomorph genome data, Orf222 was identified as being homologous to a number of proteins including Slr1649 from *Synechocystis *sp. PCC 6803 and CpcT from *Synechococcus *sp. PCC 7002. Because cryptophytes are inaccessible to genetic manipulations, we created a *slr*1649-loss-of-function strain of *Synechocystis *sp. PCC 6803 and complemented this strain with the nucleomorph-encoded orf.

The generated Slr1649 loss-of-function mutant generally has characteristics conductive with the description by Shen et al. for a *cpcT *knock-out in *Synechococcus *sp. PCC 7002 [10]. Nevertheless, additional effects in the *slr*1649 knock-out mutant of *Synechocystis *sp. PCC 6803 were identified in respect to linker proteins within the phycobilisomes of the mutant. Complementation of *slr*1649 with the nucleomorph-specific *orf*222 indicated that the cryptophytic protein, although having originated from an organism using phycoerythrin as accessory pigment, attaches a bilin to the position Cys-β155 of phycocyanin in the cyanobacterium.

## Results

### *In silico *analyses

After analyzing the nucleomorph genome of the cryptophyte *Guillardia theta*, we identified an open reading frame (*orf*222) with a high degree of similarity to cyanobacterial genes (Table 1), that encode soluble proteins possessing a DUF1001 domain. Alignments of the cryptophyte sequence with these cyanobacterial sequences indicated that *orf*222 should encode an additional transit peptide as shown by a N-terminal extension. Further orfs with homology to *orf*222 and the cyanobacterial homologs are additionally present in the nuclear genome of red alga [24]. In higher plants, i.e. *Arabidopsis thaliana*, orfs with some similarity are also present [25]. Even the bacteriophage S-PM2, which infects *Synechococcus *strains, encodes a homolog of *orf*222 [26] (Table 1).

**Table 1:** Tabular comparison of prokaryotic and eukaryotic homologous of Orf222

**Organism**		**PBP**	**Homologous of Orf222**	**Length (aa)**	**CpeT homolog**	**Slr1649 homolog**	**Genomic context of the encoded genes**	
*Synechocystis*	S. sp. PCC 6803	PC	Slr1649	196			*slr1648/ssr2754/****slr1649***	

*Crocosphaera watsonii*	WH 8501	PE	CwatDRAFT_4238	196		x	***cwatDRAFT_4238****/cwatDRAFT_4297*	
		PC	CwatDRAFT_0664	215	x		*cpeA****/cpeT****/cpeY*	
			CwatDRAFT_5720	149	x		*cpeS*/***cpeT***/*cpeR*	

*Nostoc*	*Nostoc punctiformes *PCC 73102	PEC	Npun02004130	197		x	*thrC/****npun02004130****/npun02004132*	
		PC	Npun02004123	209	x		*cpeS/****cpeT****/cpeR*	
			Npun02007740	201	x		-	
	*Anabaena *sp. PCC7120		All5339	199		x	-	
			Alr0647	198	x		-	
	*Anabaena variabilis *ATCC 29413		Ava_2579	199		x	-	
			Ava_4579	198	x		-	

*Thermosynecho-coccus elongatus*	BP-1	PC	Tlr2156	196		x	*tlr2154*/*hemD/****tlr2156***	

*Synechococcus*	S. elongates PCC 6301	PC	Syc0738_d	197		x	***syc0738_d****/syc0739_d/ruvC*	
		PE	Syc0764_d	197	x		-	
	S.sp. PCC 7002		CpcT	199			from [10]	
	S.sp. CC 9311		Sync_0487	196		x	*sync_0484/cpcF/cpcE/****sync_0487****/cpcA/cpcB/pebB*	
			Sync_0509	208	x		*cpeC/sync_0512/cpeD-1/cpeS/****sync_0509***	
	S. elongates PCC 7942		Synpcc7942_0772	197	x		-	
			Synpcc7942_0800	197		x	***synpcc7942_0800****/synpcc7942_0799/ruvC*	
	S. sp. CC 9605		Syncc9605_0440	204	x		*cpeS/****cpeT****/cpeR*	
			Syncc9605_0419	208		x	*cpcB/cpcA/****syncc9605_0419****/phycocyanobilin- lyase*	
	S. sp. WH 8102		SYNW2024	197		x	*rpcB/rpcA/****synw2024****/phycocyanobilin-lyase*	
			SYNW2003	204	x		*cpeC/mpeD/cpeE/cpeS/****cpeT****/cpeR*	
	S. sp. CC9902		Syncc9902_1910	200		x	*cpcB/cpcA/****9902_1910****/phycocyanobilin-lyase*	
			Syncc9902_1887	204	x		*cpeS/****cpeT****/cpeR*	

*Trichodesmium erythraeum*	IMS101	PC	Tery_0543	195		x	*cyp/cyp/****tery0543****/transposase*	
		PE	Tery_0979	209	x		*cpeZ/****cpeT****/cpeF*	

*Calothrix*	PCC 7601	PC	CpeT	207	x		*cpeS/****cpeT****/cpeR*	
		PE						

*Gloeobacter Violaceus*	PCC 7421	PE	Glr1182	202			*apcD/****glr1182****/cpcB/cpcA*	
		PC	Glr1193	203	x		*cpeS/****cpeT***	
			Glr1538	183	x		-	

*Prochlorococcus marinus*	MIT 9211	PE	P9211_07167	192	x		*cpeS/****cpeT***	All adjacent to the α- and β- subunit of phycoerythrin
	CCMP1375		Orf195	195	x		*cpeS/****cpeT***	
	MIT 9313		PMT1678	239	x		*cpeS/****cpeT***	
	NATL2A		PMN2A_1676	199	x		*cpeS/****cpeT***	
	SS120		Pro0342	195	x		*pro0347/pucC/ppeC/pro0344/cpeS/****cpeT***	

*Guillardia theta*		PE	Orf222	222				

*Cyanidioschyzon merolae*		PC	CMK263C	263				

Bacteriophage *S-PM2*	host = Synechococcus		S-PM2p215	175				

*Oryza sativa*			LOC_Os11g32160	275				

*Arabidopsis thaliana*			AT5G51020	269				

Cyanobacterial and eukaryotic homologous of Orf222 were obtained by blast search analysis of the NCBI database, as well as the JGI and Cyanobase. The (-) indicates that no adjacent genes in the same orientation were detectable. Orf222 homologous genes are in bold. PBP = phycobiliproteins (except of allophycocyanin), PC = phycocyanin, PE = phycoerythrin, PEC phycoerythrocyanin.

**Figure 1:**
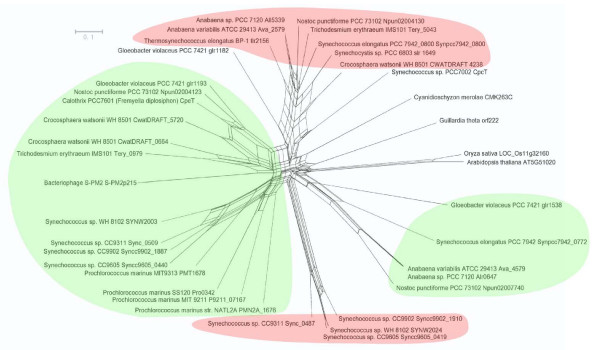
**NeighborNet (NNet) splits graph for 41 taxa**. Proteins sequences were aligned with MUSCLE. The initial alignment contained 307 sites including 191 gapped sites that were excluded from the analysis, leaving 116 amino acid sites for log determinant (LogDet) distance estimates with removal of invariant sites using the program LDDist. From this a Neighbor – net splits graph was constructed, which is visualized with Splitstree4. Highlighted are four monophyletic groups: Two of them resemble CpeT-like (phycoerythrin operon protein) proteins (highlighted in green) and two groups harbor members of the Slr1649-like type (highlighted in red). Not shown: The sequences of *Synechococcus elongatus *PCC 7942 Synpcc7942_0800 and *Synechococcus elongatus *PCC 6301 Syc0738_d are identical as well as the sequences of *Synechococcus elongatus *PCC 7942 Synpcc7942_0772 and *Synechococcus elongatus *PCC 6301 Syc0764_d and the sequences of *Prochlorococcus marinus *SS120 Pro0342 and *Prochlorococcus marinus *CCMP1375 orf195.

The number of Orf222 homologues in cyanobacteria varies in several species and does not correlate with the number of phycobiliproteins. Nevertheless, there is a strong tendency to express more than one species of Orf222 homolog in organisms containing multiple types of phycobiliproteins in the rods (Table 1). Based on amino acid sequence alignments and phylogenetic networks, four monophyletic groups can therefore been assigned (Fig. 1). Two of them resemble CpeT-like proteins (phycoerythrin operon protein); the other two groups harbor members of Slr1649-like type. Neither the *Guillardia theta *sequence nor any other eukaryotic sequences can be assigned to any one of the four monophyletic groups.

Additionally, clear affiliations of the *Gloeobacter violaceus *PCC 7421 (glr1182) and *Synechococcus sp*. PCC 7002 (CpcT) sequences can not be extrapolated. With the exeption of the *Prochlorococcus *species, at least one member of the Slr1649-like group is present in all cyanobacteria investigated to date. CpeT-like proteins were only detected in cyanobacteria encoding phycoerythrin and/or phycoerythrocyanin. Although the proteins of both groups seem to have the same function, further investigations on the corresponding genes relevant in the genomic context revealed a noticeable difference. Unlike the genes of the *cpeT*-group, the *slr*1649-group is by far less conserved in its genomic localization (Table 1). Except for *Nostoc *sp. PCC 7120, *Anabaena variabilis *ATCC 29413 and *Trichodesmium erythraeum *IMS 101, the localization of the homolog gene is always downstream of *cpeS*. In few cases it is followed by *cpeR*. On the other hand, genes for the *slr*1649-group are rather randomly distributed in the investigated cyanobacterial genomes (Table 1).

### Generation of a *slr1649 *knock-out strain

We used *Synechocystis *sp. PCC 6803 as a model organism and created first a *slr*1649 knockout strain (Δ*slr1649*) by inserting a kanamycin resistance cassette into the *slr*1649 open reading frame via homologous recombination. The generated homozygous knock-out mutant showes identical features described in Shen et al. [10]. Just like the characterized *cpcT *mutant in *Synechococcus *sp. PCC 7002, our knock-out mutant contains a decreased level of phycocyanin up to 60% and a resulting pale green phenotype. The knock-out cells produce smaller phycobilisomes, which could be the cause of their different migration behaviour in sucrose density gradients in comparison to wild type phycobilisomes. Furthermore, isolated phycobilisomes showed a red-shifted absorbance maxima and a slightly smaller apparent molecular mass in the β-subunit of phycocyanin on SDS-PAGE (data not shown). After the digestion of purified phycocyanin with formic acid and a phycocyanobilin addition assay, Shen et al. concluded after digestion that the *cpc*T gene from *Synechocccus *sp. PCC 7002 encodes a bilin lyase responsible for the attachment of phycocyanobilin to Cys-153 on the β-subunit of phycocyanin [10]. The same is most likely true for the *Synechocystis *homolog Slr1649 due to the high homology and the similar phenotype between the two knock-out mutants. Thus, Slr1649 is thought to attach a bilin group to the homolog position Cys-155 of β-phycocyanin in *Synechocystis *sp.PCC 6803.

### Isolation and analysis of phycobilisomes

**Figure 2:**
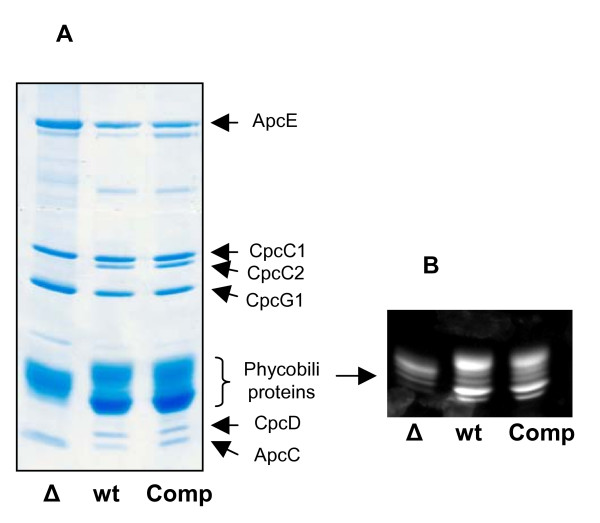
**SDS-PAGE of phycobilisomes isolated from sucrose gradients**. (A) Coomassie staining of separated phycobilisome components from Δ*slr1649 *(Δ), wild type and complemented (Comp) strains. Both linker proteins (CpcC2 and CpcD), which are absent in Δ*slr1649 *cells, are present in the complemented strain, indicating a wild type phycobilisome structure. The linker proteins CpcC2 and CpcD are absent in the mutant strain (Δ). (B) Zn^2+ ^staining of phycobilisomes separated in SDS-PAGE. The signal intensity of phycobiliproteins isolated from wild type and complemented (Comp) strains seems to be equal in contrast to the reduced signal of the Δ*slr1649 *mutant.

By isolating and analysing phycobilisomes from our knock-out mutant, we observed one additional feature besides the one already known from the characterization of the *Synechococcus *sp. PCC 7002 homolog. As shown in Fig. 2A two linker polypeptides, CpcC2 and CpcD, encoded by the genes *cpcC2 *and *cpcD *respectively, are missing in the isolated phycobilisomes of the mutant but could be identified in the wild type during mass spectrometry analysis. The other linker polypeptides CpcC1 (*cpcC1)*, CpcG1 (*cpcG1*), ApcC (*apcC*) and ApcE (*apcE*) are present in both strains.

Upon further investigations, the absence of the linker proteins was proven not to be a transcriptional effect. In exemplarily reverse transcription experiments for *cpcC2*, the presence of identical *cpcC2 *transcripts was confirmed in both the mutant and the wild type strain by sequencing the obtained RT-PCR products (data not shown).

### Complementation

**Figure 3:**
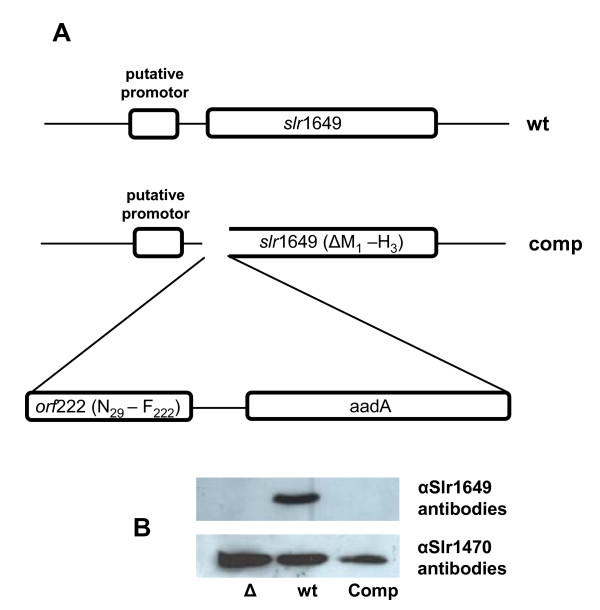
**Complementation construction and control experiments**. (A) Schematic picture of the complementation construct. Schematic depiction of the construct used for the complementation of *slr*1649 with the cryptophytic *orf*222. The upper figure displays the wild type situation. The lower figure shows the insertion site of *orf*222, without its putative transit peptide and the *aadA *gene into *slr*1649 by simultaneously affecting the reading frame of *slr*1649 and its *cis*-acting upstream signals. (B) Immunoblot with Slr1649 (upper) and Slr1470 (lower) specific antibodies. Cells from Δ*slr1649*, wild type and complemented strains were disrupted and the protein extracts were separated by SDS-PAGE. Neither in the fraction of Δ*slr1649 *cells nor in the fraction of the complemented strain were signals of the Slr1649 antibody detectable. Specific polyclonal Slr1470 antibodies were used as a loading control and clear signasl at the expected size were obtained in all three protein fractions.

In order to investigate if the nucleomorph-specific reading frame *orf*222 from the cryptophyte *Guillardia theta *is able complement the effects of the *slr*1649 loss-of-function, we integrated this potential gene without its putative transit peptide into the cyanobacterial genome of *Synechocystis *sp. PCC 6803. This simultaneously affected the reading frame of *slr*1649 and its *cis*-acting upstream signals (Fig. 3A). In the complemented strain, *slr*1649 is separated from its natural upstream region, generating a promoter-less truncated gene, in which the translational initiator codon and the next two codons are no longer present in the reading frame. The loss of the *slr*1649 gene product and the complete segregation of the mutation were shown by immunoblot experiments using polyclonal antibodies generated against Slr1649 (Fig. 3B). Here, cross-reactions of the antibody were shown to be present in the wild type but not in the mutant strain extract. Additional analysis of the complemented strain by RT-PCR and sequence analysis showed that the integrated cryptophytic *orf*222 is transcribed (data not shown).

### Characterization of the complemented strain

**Figure 4:**
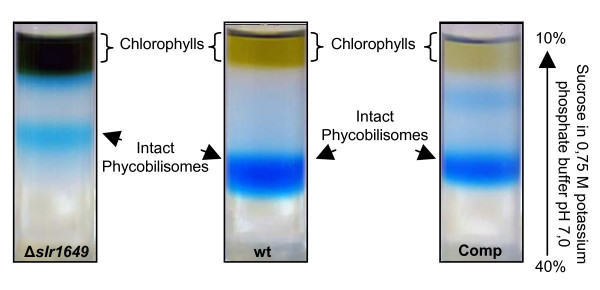
**Isolation of intact phycobilisomes**. Phycobilisomes were isolated as described in Material & Methods. After 16 h centrifugation the phycobilisomes became visible as clear blue bands in the gradient. The upper layer contained chlorophylls. The phycobilisomes of Δ*slr1649 *cells had a diminished migration compared to the wild type ones, whereas the phycobilisomes from the complemented strain (Comp) had a migration equivalent to the wild type.

Interestingly the phenotype of the complemented strain is similar to that of the wild type strain, as indicated by the greenish color of the culture (data not shown). During sucrose density-gradient separation of isolated phycobilisomes from the complemented strain, we noticed no difference in the migration behaviour of the prominent band in respect to the wild type strain in contrast to the migration behaviour of the knock-out mutant (Fig. 4). This indicates that the size of the phycobilisomes is identical in both strains and could indeed be confirmed by a proteome and mass spectrometry analysis. Here we showed that the missing linker proteins of the *slr*1649 knock-out strain were present in the complemented strain (Fig. 2A). Additionally, there was no molecular mass shift in the β-subunit of phycocyanin of the complemented strain on SDS-PAGE visible. Further analyses revealed that the chromophore group, missing at positions Cys-β153 and Cys-β155 in the knock-out mutants of *Synechococcus *sp. PCC 7002 and *Synechocystis *sp. PCC 6803 respectively, most probably reappeared in the complemented strain, because Zn^2+ ^stainings of phycobilisomes separated by SDS-PAGE showed an equal signal intensity of the phycobiliproteins of the wild type and complemented strain (Fig. 2B).

**Figure 5:**
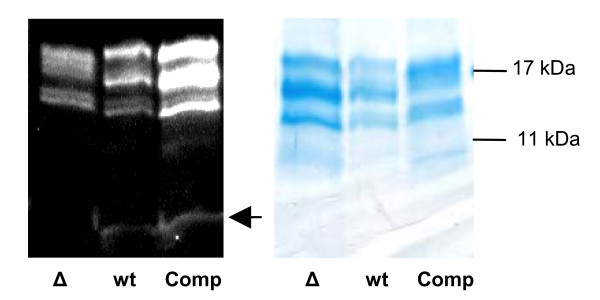
**Proteolytic digestion of phycobilisomes**. (A) Digestion of phycobilisomes with formic acid. The arrow indicates the resulting fragment after Zn^2+ ^stain at 2.78 kDa. There are also several signals at 17–20 kDa which refer to the unaffected α-subunit of phycocyanin and the α- and β-subunit of allophycocyanin.

To clarify this we digested isolated phycobiliproteins with formic acid. In doing so, CpcB is cleaved at a single site while all other phycobiliproteins remain unaffected. The expected sizes for fluorescent fragments are 15.36 kDa with a chromophore group at position Cys-β84 and 2.78 kDa for the fragment with the chromophore group at position Cys-β155. As shown in Figure 5, these expected fragments were obtained. In addition to a signal at 15.36 kDa, a signal at 2.78 kDa was detected in the lane containing wild type strain protein and protein from the complemented strain but not in the one containing Δ*slr1649 *protein. These data confirmed that the chromophore group, missing in the knock-out mutant, is present in the complementation. This together with the identification of the linker protein spectrum in the complemented strain indicated a wild type phycobilisome structure.

## Discussion

Cryptophytes are important organisms for several reasons. In terms of cell biology, their complex compartmentalization is of major interest, because several plasmas and genomes coexist in these organisms, which can be traced back to either a prokaryote or a eukaryote [27]. One of the hallmarks of cryptophytes is the remnant of a second nucleus, which originated by the reduction of the cell nucleus of an engulfed phototrophic eukaryote by another eukaryotic cell [28]. This compartment, the nucleomorph, is minimized in its coding capacity and expresses – in the case of *Guillardia theta *– only approximately a tenth of that of the *E. coli *K12 genome [29]. The reduced coding capacity leads to the impression that the genes are still present in the nucleomorph may encode important functions. Thus, we are interested in addressing the functions of proteins encoded by the nucleomorph. However, due to the lack of a method of transfecting cryptophytes, we are studying homologs of the nucleomorph genes and their encoded proteins in genetically accessible organisms in order to identify the functions of the cryptophytic proteins indirectly. One of the best-studied and genetically accessible cyanobacterium is *Synechocystis *sp. PCC 6803.

Orf222 is one of the uncharacterized nucleomorph-specific open reading frames, for which homologs are present in many cyanobacteria. Analysis of the contribution of this gene within different organisms indicated that a clear correlation between *orf*222-homolog genes and phycobiliproteins is present, because at least one *orf*222 homolog is encoded in all organisms expressing phycobiliproteins, including red alga. Phylogenetic studies demonstrated that homologs of the *orf*222 gene can be classified into the following four groups (Fig. 1): Slr1649-like a, Slr1649-like b, CpeT-like a and CpeT-like b. Because the method for network construction as well as sampling in our studies is different from that of a recently presented phylogeny [10], it is not surprising that slightly different affiliations are resolved. However, our network corrects erroneous affiliations and indicates uncertainties of the basal grouping. This may be seen in the position of the bacteriophage sequence, which is in the network presented here in the neighbourhood of the bacteria they infect and not in the same branch as the cryptophyte sequence.

Despite the high degree of homology, the members of Slr1649-like and CpeT-like groups differ in the genomic context of the corresponding genes (Table 1). Members of the CpeT group are predominantly localized in the phycoerythrin associated linker protein operon [30,31] next to the *cpeS *gene. In some cases, even the *cpeR *gene is localized directly downstream of *cpeT*. Because operon structures connect functionally related genes in many cases, CpeT could be an either structurally or functionally moiety of the phycobilisome and it has been shown to be responsible for the attachment of a bilin group to a specific site from β-phycocyanin [10]. It is remarkable that a congruent distribution of members of the Slr1649-groups is not visible, because the genes seem to be localized randomly throughout different genomes. Interestingly, *slr*1649-homologs exist in some higher plants such as *Oryza sativa *and *Arabidopsis thaliana *(Fig. 1). The encoded proteins of these land plants are characterized by a DUF1001 domain as well, but obviously have paralogous functions, since the *Arabidopsis thaliana *homolog seems to be required for plastid division [25]. It is also suggested to play an important role in cell differentiation and the regulation of the cell division plane in plants [25]. The same could be true for the copy of the bacteriophage S-PM2, but seems to be unlikely since this phage infects different *Synechococcus *strains and its resource of the homolog may be the result of a selective advantage.

The homozygous knock-out mutant Δ*slr1649 *in *Synechocystis *sp. PCC 6803 showed features identical to a *cpcT *knock-out mutant from *Synechococcus *sp. PCC 7002 described in Shen et al. [10]. Here, the same pale green phenotype and a reduced phycocyanin content, resulting from a missing bilin group in phycobilisomes, was created by knock-out of *cpc*T, homolog to *slr*1649 homolog in this cyanobacterium. This indicates that the lyase function of the homologous proteins of *Synechococcus *sp. PCC 7002 and *Synechocystis *sp. PCC 6803 is comparable.

Nevertheless, we obtained one additional, not described feature in the *Synechocystis *sp. PCC 6803 knock-out mutant. Two linker proteins, CpcC2 and CpcD, were missing from the phycobilisomes in the knock-out mutant Δ*slr1649*. CpcD is a small linker (10 kDa) located at the distal tip of rods, possibly functioning as a rod terminating factor [32]. The CpcC2 rod linker (30 kDa) connects the most distal located phycocyanin discs [33]. Both genes are located in the phycocyanin operon from which they are co-transcribed with the phycocyanin subunits and the *cpcC1 *linker gene [33]. A transcriptional effect causing the loss of the linker proteins appears to be very unlikely, because the α-subunit, the β-subunit and CpcC1 linker are present, although the CpcD and the CpcC2 linker are simultaneously absent. This is indicative of our finding that the *cpcC2 *gene is indeed transcribed in the mutant as indicated by reverse transcription experiments (data not shown). Therefore, the deficit of the two linker proteins in mutant phycobilisomes is a post-transcriptional effect. However, we can not rule out that a decreased stability of phycobilisomes caused by the altered β-phycocyanin may be the reason for the lack of the two linker proteins in our preparations. In any case, the lack of the linker proteins is a molecular marker for the loss of lyase function, which may be interested to be studied in *Synechococcus *sp. PCC 7002 [10] as well.

*Guillardia theta*, the cryptophyte on which we are primarily focusing expresses a homolog of *slr*1649 in association with phycoerythrin. Phycobiliproteins are located in the thylakoid lumen and apparently not organized in phycobilisomes in cryptophytes. Because the cryptophyte *Guillardia theta *uses phycoerythrin and not phycocyanin as an accessory pigment for photosynthesis, one might not expect that the putative cryptophytic lyase is able to complement the one of *Synechocystis *sp. PCC 6803. Surprisingly, the complemented strain showed wild type phycobilisomes structures as shown by the correct attachment of chromophore groups and the linker protein spectrum. Thus, Orf222 from the cryptophyte is able to complement the loss-of-function of Slr1649, indicating that the cryptophytic phycoerythrin lyase has still retained the capacity to couple a bilin group to β-phycocyanin, even after the progenitor of both classes of proteins evolved into apparently paralogous ones. However, a pleiotropic function of a biliprotein lyase with a specificity for phycobilin:cysteine-84 was recently shown *in vitro *for CpeS1 from *Anabaena *PCC 7120 [11], implicating that a multiplicity of proteins like the cryptophytic phycobilin:cysteine-β155 lyase has the capacity to couple bilins to homologous positions in a variety of phycobiliproteins.

## Conclusion

Loss-of function of a bilin lyase leads to a variety of effects in phycobilisome structure. This is already shown for a *cpcT *mutant in *Synechococcus *sp. PCC 7002 and could be confirmed by the generation of a *slr*1649 knock-out mutant in *Synechocystis *sp. PCC 6803, homolog *cpcT*. One additional feature, the lack of two distal linker proteins, fits with the already known altered phycobilisome structure and may be the reason for the decreased phycocyanin content in mutant missing the bilin lyase.

Loss of Slr1649 was complemented *in vivo *by the homolog Orf222, which is encoded by the tiny vestigial nucleus of the eukaryotic endosymbiont from the cryptophyte *Guillardia theta*. Thus, Orf222 is supposed to be a phycoerythrin-bilin lyase in cryptophytes. Despite having originated from an organism using phycoerythrin as its accessory pigment, the protein still has the capacity to couple a chromophore group to the β-subunit of phycocyanin, indicating the functional universality of bilin lyases on the one hand and demonstrating the importance of nucleomorph-encoded cellular functions on the other.

## Methods

### Cell Culture

*Synechocystis *sp. PCC 6803 strains, wild type, Δ*slr1649 *and the complemented strain, were grown at 30°C in Erlenmeyer flasks containing BG-11 media [34] with gentle swirling under standard light conditions (70 μE) and atmospheric CO_2 _levels. For growth on plates, BG-11 medium was supplemented with 1% Agar. Plates were incubated under the same conditions as liquid cultures.

### Construction of the Δ*slr1649 *Mutant

Two pairs of primers were used to amplify the flanking regions of the knock-out construct: 1649a_f (5'-GGT TAC TGC TCG AGG CGC ATC A-3') and 1649a_r (5'-GGA CGG CAA GGG ATC CTA TCT GG-3') generate fragment slr1649a, 1649b_f (5'-GGA CGG CAA GGG ATC CTA TCT GG-3') and 1649b_r (5'-CAG AAA TTG CCG CGG CCA ATC TC-3') fragment 1649b. Both were ligated into the pGEM-T vector (Promega, Mannheim) and after verification of the sequence, transferred into the pBluescript II SK (Stratagene, Amsterdam) vector. *Escherichia coli *strain MRF' XL-1 blue was used as plasmid host for cloning steps.

Using the *Bam*HI restriction site (inserted by the primer 1649a-r and 1649b_f), a kanamycin resistance gene was cloned between the two fragments resulting in plasmid pΔ1649. After transforming into wild type *Synechocystis *sp. PCC 6803 cells with this plasmid, transformants were selected on BG-11 agar plates supplemented with kanamycin (5 μg/ml starting concentration). Kanamycin resistant clones were transferred to BG-11 liquid media. A homozygous culture was achieved by increasing kanamycin concentrations (50 μg/ml final concentration). Complete knock-out was confirmed via Southern blot analysis.

### Construction of the Complemented Strain

Nucleotide sequence of *orf*222 was amplified from *Guillardia theta *DNA without its putative transit peptide by using the primers 222komp2_f (5'-CAT ATG AAT TAA AAC CAA TCC TTA ATT G -3') and 222komp_r (5'-GTT AAA ATT AAA TGA ATT CTA ATA A-3'). Two pairs of primers were used to amplify the flanking regions: 1649a_f (5'-GGT TAC TGC TCG AGG CGC ATC A-3') and 1649kompa1_r (5'-CAA TAA CTA CAT ATG TCC CAT TCC-3') generated the fragment Compa, which includes the upstream region for *slr*1649, 1649kompa2_f (5'-TTT ATG TCG AAT TCC ACT GAT C-3') and 1649b_r (5'- GAG ATT GGC CGC GGC AAT TTC TG-3') generated fragment Compb. All three fragments were ligated into the pGEM-T vector (Promega, Mannheim) and after verification of the sequence, transferred into the pBluescript II SK (Stratagene, Amsterdam) vector using different restrictions sides inserted by the primers leading to a precursor construct. By using *Eco*RI restriction sites, a spectinomycin cassette was cloned between the two fragments Compa/*orf*222 and Compb resulting in plasmid pComp222 Δ*slr*1649. After transforming the *Synechocystis *sp. PCC 6803 wild type strain with this plasmid, transformants were selected on BG-11 agar plates supplemented with spectinomycin (5 μg/ml starting concentration). Spectinomycin resistant clones were transferred to BG-11 liquid media. A homozygous culture was achieved by increasing spectinomycin concentrations (30 μg/ml final concentration).

### Nucleic Acid Analysis

*Synechocystis *sp. PCC 6803 cells were collected by centrifugation of 5 ml cell culture at 3200 × g. For DNA isolation, the pellet was resuspended in 400 μl TE buffer pH 7.0. After addition of breaking buffer (10% sodium dodecyl sulfat (w/v), 5% sodium lauryl sulfat (w/v)), 200 μl glass beads (0.2 mm diameter) and 400 μl phenol, cells were lysed by vortexing the suspension three times for 10 s. The suspension was then centrifuged at 12 000 × g and the resulting upper phase transferred to a new cup. This sample was treated twice with phenol-chloroform-isoamylalcohol (25:24:1) and centrifuged as before. By adding 1/10 Vol. NaAc pH 4.8 and two Vol. 96% ethanol, the DNA was precipitated for 1 h at -20°C. Afterwards, an additional washing step with 70% ethanol was performed. The pellet was dried and resuspended in H_2_O.

RNA was isolated from *Synechocystis *cells with Trizol^© ^(Invitrogen, Karlsruhe) according to the manufacturer protocol. Northern Blot and Southern Blot analysis were performed according to standard protocols (Sambrook). Probes were constructed using the PCR DIG Probe Synthesis Kit (Roche, Mannheim).

### Antibody Generation and Purification

To generate an antibody against Slr1649 we used the primers ex1649_f (5'-GGA TCC TTA TGT CCC ATT CCA CTG-3') and ex1649_r (5'-CTC GAG GCT GGC TAA AAA CTA ACT-3') to amplify the *slr*1649 gene, which was finally cloned in the pGEX-5X-3 vector (GE Healthcare Biosciences). After overexpression and purification of the Slr1649 GST fusion protein, immunization steps were executed by the Eurogentec company (Seraing).

The IgG fraction was purified from serum by protein A sepharose beads (GE Healthcare Biosciences).

### Isolation of Phycobilisomes

Phycobilisome isolation was performed according to Gray et al. [35]. Cells were collected by centrifugation at 5000 rpm for 10 min at room temperature. After an additional washing step with BG-11 media, cells were resuspended in 0.75 M potassium-phosphate buffer pH 7.0 (PPB), containing a protease inhibitors cocktail (PIC, 2 mg/ml Antipain, 5 mg/ml Chymostatin, 2 mg/ml Aprotinin, 5 mg/ml Trypsin-Inhibitor, 2 mg/ml Pepstatin, 5 mg/ml Leupeptin, 1 mg/ml Elastatinal and 2 mg/ml Na_2_EDTA in HEPES/KOH. Final concentration 200 μg/ml Inhibitor) and afterwards broken by two passes through a French press (Aminco) at 124 MPa. The lysates were incubated with Triton X-100 (2%) for 15 min at room temperature and subsequently centrifuged at 20 000 rpm for 1 h to pellet unbroken cells and membrane debris. The supernatant was immediately loaded on a 10%–40% linear sucrose gradient, solved in PPB and centrifuged at 18 000 rpm for 16 h at 15°C.

### SDS-PAGE

Standard SDS-PAGE was performed with an Hoefer SE 250 apparatus (83 mm × 101 mm, 0,75 mm thick) or a custom made system (250 mm × 150 mm and 1,0 mm thick) using the Laemmli buffer system [36]. The polyacrylamide content in the separating gel was a gradient of 10% to 15%. The stacking gel contained 6% polyacrylamide. To achieve a better resolution of polypeptides with masses less than 15 kDa, the SDS-Tricine gel system was used [37]. Staining of gels was generally carried out with Coomassie brilliant blue G-250 dissolved in solution A (2% phosphoric acid v/v, 10% (NH_4_)_2_SO_4 _w/v) and methanol (40:9:1). To visualize the bilin carrying proteins gels were incubated in a 0.2 M ZnSO_4 _solution [38,39] and highlighted with UV in a transilluminator (Bio-Rad).

### Formic Acid Cleavage

Phycobilisomes were precipitated with Methanol/Chloroform [40] and resuspended in cleavage buffer. Cleavage was done according to Piszkiewicz et al. [41]. 30 μg of phycobilisomes were incubated for 16 h at 37°C with 70% formic acid in before adding SDS sample buffer and analysis by Tricine SDS-PAGE on a 17% polyacrylamide gel.

### Isolation of Protein Extracts from *Synechocystis *sp. PCC 6803

*Synechocystis *sp. PCC 6803 cells were grown and harvested as described above. The cell pellet was resuspended in TEN100 buffer [42], containing PIC. Cell lysis was performed as described above.

### MALDI-TOF MS Analysis

The protein spots were subjected to in-gel trypsin digestion before mass spectrometry analysis as described previously [43]. The peptide mixtures from the tryptic digests were desalted and concentrated using ZipTips™ columns made from the reverse chromatography resins Poros and Oligo R3 (Applied Biosystems). The bound peptides were washed with a solution of 0.5% formic acid and eluted from the column in 1 μl of 33% (v/v) acetonitrile/0.1% trifluoroacetic acid solution saturated with α-cyano-4-hydroxycinnamic (Bruker Daltonics) directly onto a MALDI target plate and air dried before analysis in the mass spectrometer. Mass spectrometry measurement was performed on an Ultraflex-TOF TOF tandem mass spectrometer (Bruker Daltonics). Peptide mass fingerprint spectra were acquired in the reflectron positive mode with a pulsed extraction using approximately 100 laser shots. The spectra were acquired after an external calibration using reference peptides (Peptide mixture II Bruker Daltonics). The acquired spectra were further internally calibrated using trypsin autolysis peaks as internal standards (842.5100, 2211.1046 Da). Monoisotopic masses were assigned and processed using Biotools ™ and FlexAnalysis ™ software (Bruker Daltonics) before submitting them to the Mascot program [44] for searches against the non-redundant NCBI database. The parameters used in the Mascot peptide mass fingerprint searches were as follows: Taxonomy, *Synechocystis*; search all molecular masses and all isoelectric points; allow up to one missed proteolytic cleavage site and a peptide mass tolerance of 50 ppm. Methionine oxidation was considered as an optional modification and cysteine carbamidomethylation as a fixed modification in all the searches. Matches to *Synechocystis *proteins were considered unambiguous when the probability score was significant using the Mascot score with a p value < 0.05 and when there was a minimum of five peptides and with a sequence coverage greater than 20%. For each protein the identity was further validated by tandem MS-MS analysis of selected peptides.

### *In silico *Analysis

Blast search analyses were done by NCBI protein-protein blast [45] (see Additional file 1). This program was also used for conserved domain predictions. Transmembrane domains were predicted by using TMHMM server v. 2.0 [46] and the SOSUI protein prediction server [47]. Genome data from *Synechocystis *sp. PCC 6803 were obtained from CyanoBase [48]. *In silico *cleavage was performed by PeptideMass [49].

### Network Construction

The 41 sequences were aligned using MUSCLE [50] with 16 iterations. The output format was set to the standard ClustalW format [51]. The alignment contained 307 sites including 191 gapped sites that were excluded from the analysis. From the remaining 116 sites logdet distances were estimated using LDDist 1.3.2 [52]. These distances were used to construct a Neighbor-net splits graph [53] that was visualized with SplitsTree4 [54].

## Authors' contributions

KB, OK and JP performed the experiments except phylogenetic NG and MALDI-TOF analyses JN, UGM initiated the project and was the supervisor. The manuscript, to which all authors contributed, was designed and written by KB, OK and UGM All authors have read and approved of the final version of the manuscript.

## Supplementary Material

Additional file 1 **Accession numbers**. Sequences were obtained from NCBI and the *Cyanidioschyzon merolae *Genome Project with the exception of cpcT from *Synechococcus *sp. PCC 7002, which was downloaded from [10]. Click here for file
