# Supplementary material for: Complementation of a phycocyanin-bilin lyase from Synechocystis sp. PCC 6803 with a nucleomorph-encoded open reading frame from the cryptophyte Guillardia theta
Source: BMC Plant Biol. 2008 May 16;8:56. doi: 10.1186/1471-2229-8-56 (PMC2408579; doi:10.1186/1471-2229-8-56)
Supplement: Additional file 1 — Accession numbers. Sequences were obtained from NCBI and the Cyanidioschyzon merolae Genome Project with the exception of cpcT from Synechococcus sp. PCC 7002, which was downloaded from [10]. [file 1471-2229-8-56-S1.pdf]

Accession numbers

Sequences were obtained from NCBI and the *Cyanidioschyzon merolae* Genome Project with the exception of cpcT from *Synechococcus* sp. PCC 7002, which was downloaded from [10]. *Synechocystis* sp. PCC 6803: Slr1649 [GenBank:NP_441787], slr1648 [GenBank:BA000022], ssr2754 [GenBank:BA000022], ssr2755 [Genbank:]; *Crocosphaera watsonii*: CwatDRAFT_4238 [GenBank:ZP_00515713], CwatDRAFT_0664 [GenBank:ZP_00518424], CwatDRAFT_5720 [GenBank:ZP_00514725], CwatDRAFT_4297 [GenBank:AADV02000007], CwatDRAFT_5719 (cpeS) [GenBank:ZP_00514726], CwatDRAFT_5721 (cpeR) [GenBank:ZP_00514724], CwatDRAFT_0663 (cpeA) [GenBank:ZP_00518423], CwatDRAFT_0665 (cpeY) [GenBank:ZP_00518425]; *Nostoc punctiformes* PCC 73102: Npun02004130 [GenBank:ZP_00109088], Npun02004123 [GenBank:ZP_00109082], Npun02007740 [GenBank:ZP_00106171], Npun02004134 [GenBank:ZP_00345510], Npun02004129 (thrC) [GenBank:ZP_00109087], Npun02004122 (cpeS) [GenBank:ZP_00109081], Npun02004124 (cpeR) [GenBank:ZP_00109083]; *Anabaena* sp. PCC 7120: all5339 [GenBank:NP_489379], alr0647 [GenBank:BAB72605]; *Anabaena variabilis*: Ava_2579 [GenBank:YP_323089], Ava_4579 [GenBank:YP_325071]; *Thermosynechococcus elongates* BP-1; tlr2156 [GenBank:NP_682946], tlr2154 [GenBank:BAC09706], tlr2155 (hemD) [GenBank:NP_682945]; *Synechococcus elongates* PCC 6301: syc0738_d [GenBank:YP_171448], syc0764_d [GenBank:YP_171474], syc0740_d (ruvC) [GenBank:Q5N440], syc0739_d [GenBank:BAD78929]; *Synechococcus* sp. CC9311: sync_0487 [GenBank:ABI46022], sync_0509 [Genbank: ABI47049], sync_0484 [Genbank:ABI45763], sync_0485 (cpcF) [Genbank:ABI45480], sync_0486 (cpcE) [Genbank:ABI47764], sync_0488 (cpcA) [Genbank:ABI45378], sync_0489 (cpcB) [Genbank:ABI47294], sync_0490 (pebB) [Genbank:ABI46568], sync_0510 (cpeS) [Genbank:ABI45963], sync_0511 (cpeD-1) [Genbank:ABI47671], sync_0512

[Genbank:ABI45417], sync_0513 (cpeC) [Genbank:ABI45980]; *Synechococcus elongatus* PCC 7942: synpcc7942_0772 [Genbank:ABB56804], synpcc7942_0800 [Genbank:ABB56832], synpcc7942_0799 [Genbank: ABB56831], synpcc7942_0798 (ruvC) [Genbank:ABB56830]; *Synechococcus* sp. CC9605: syncc9605_0440 (cpeT) [Genbank: ABB34216], syncc9605_0419 [Genbank:ABB34195], syncc9605_0439 (cpeR) [Genbank:ABB34215], syncc9605_0441 (cpeS) [Genbank:ABB34217], syncc9605_0421 (cpcB) [Genbank: ABB34197], syncc9605_0420 (cpcA) [Genbank:ABB34196], syncc9605_0418 (PBS lyase HEAT-like repeat) [Genbank:ABB34194]; *Synechococcus* sp. CC9902: Syncc9902_1910 [GenBank:ABB26867], Syncc9902_1887 [GenBank:ABB26844], Syncc9902_1908 (cpcB) [GenBank:ABB26865], Syncc9902_1909 (cpcA) [GenBank:ABB26866], Syncc9902_1911 (phycocyanobilin lyase alpha subunit) [GenBank:ABB26868], Syncc9902_1912 (PBS lyase HEAT-like repeat) [GenBank:ABB26869] Syncc9902_1886 (CpeS) [Genbank:ABB26843]; *Synechococcus* sp. WH8102: SYNW2024 [GenBank:NP_898115], SYNW2003 [GenBank:NP_898094], SYNW2022 (rpcB) [GenBank:NP_898113], SYNW2023 (rpcA) [GenBank:NP_898114], SYNW2025 (putative phycocyanobilin lyase alpha and betafusion protein) [GenBank:NP_898116], SYNW1999 (cpeC) [Genbank:NP_898090], SYNW2000 (mpeD) [Genbank: NP_898091], SYMW2001 (cpeE) [Genbank: NP_898092], SYMW2002 (cpeS) [Genbank: NP_898093], SYNW2004 (cpeR) [Genbank: NP_898095]; *Trichodesmium erythraeum* IMS101: TeryDRAFT_1163 [GenBank:ZP_00674796], TeryDRAFT_1416 [GenBank:ZP_00674575], TeryDRAFT_1162 (cpyX) [GenBank:ZP_00674794], TeryDRAFT_1258 (cyp) [GenBank:ZP_00674795], TeryDRAFT_1164 (transposase) [GenBank:ZP_00674797], TeryDRAFT_1417 (cpeF) [GenBank:ZP_00674576], TeryDRAFT_1415 (cpeZ) [GenBank:ZP_00674574]; *Calothrix* PCC 7601.: cpeT [GenBank:AAK11648], CpeS [GenBank:AF334109], CpeR [GenBank:AAK11649]; *Gloeobacter violaceus* PCC 7421: glr1182 [GenBank:BAC89123], cpeT

[GenBank:BAC89134], glr1538 [GenBank:BAC89479], glr1181 (apcD) [GenBank:NP_924127], glr1183 (cpcB) [GenBank:NP_926163], glr1191 (ycf58) [GenBank:NP_926164], glr1192 (cpeS) [GenBank:NP_924138]; *Prochlorococcus marinus* MIT9313: PMT1678 [GenBank:NP_895505], PMT1677 (cpeS) [GenBank:NP_895504]; *Prochlorococcus marinus* NATL2A: PMN2A_1676 [GenBank:YP_292867], PMN2A_1677 (cpeS) [GenBank:YP_292868]; *Prochlorococcus marinus* CCMP1375: Pro0342 [GenBank:CAB52704], Pro0343 (cpeS) [GenBank:Pro0343]; *Prochlorococcus marinus* MIT9211: P9211_07167 [GenBank:ZP_01005965], P9211_07162 [GenBank:ZP_01005964]; *Prochlorococcus marinus* SS120: pro0342 (cpeT) [GenBank:NP_874736], pro0343 (cpeS) [GenBank:NP_874737], pro0344 [GenBank:NP_874738], pro0345 (ppeC) [GenBank:NP_874739], pro0346 (pucC) [GenBank:NP_874740], pro0347 [GenBank:NP_874741]; *Guillardia theta*: orf222 [GenBank:NP_113341]; *Cyanidioschyzon merolae*: CMK263C**;** Bacteriophage S-PM2: ORF216 [GenBank:CAF34280]; *Oryza sativa*: LOC_Os11g32160 [GenBank:ABA93997]; *Arabidopsis thaliana*: AT5G51020 [GenBank:NP_199915]
